# Adult Pacific Oyster (*Crassostrea gigas*) May Have Light Sensitivity

**DOI:** 10.1371/journal.pone.0140149

**Published:** 2015-10-16

**Authors:** Changlu Wu, Jiao Wang, Yanjian Yang, Zhuang Li, Ting Guo, Yongchuan Li, Xiaotong Wang

**Affiliations:** School of Agriculture, Ludong University, Yantai, 264025, China; Australian Museum, AUSTRALIA

## Abstract

Light-sensitivity is an important aspect of mollusk survival as it plays a vital role in reproduction and predator avoidance. In the Pacific oyster *Crassostrea gigas* light sensitivity has been demonstrated in the larval stage but has not yet been conclusively demonstrated in adult oysters. In this paper we describe an experiment which was undertaken to determine if adult Pacific oysters were sensitive to light. One LED flashlight was used to shine light onto adult oysters while they were filtering seawater through their shell openings. We found that the degree of opening increased gradually during the light period but rapidly decreased when the flashlight was turned off in the treated group but not in the control group. These results suggest that adult Pacific oyster may be sensitive to light.

## Introduction

Until now, four classes of mollusca (Gastropoda, Bivalvia, Polyplacophora and Cephalopoda) have been found to possess light sensitive organs [1]: chitons (Mollusca, Polyplacophora) possess ‘ocellus eyes’, with these being set dorsally in the outermost layer of the shell [2]; gastropods (snails and slugs) have a number of different eye types [3]; cephalopods (nautilus, squid and octopus) possess eyes of high morphological and functional complexity [4] with excellent perception and visual acuity [5]. Bivalves, in which light sensitive organs have also been widely studied, have two main types: structured and non-structured.

Bivalves having structured light sensitive organs include scallops [3], *Ctenoides floridanus* [6], *Arcanoae* [7], *Tridacna gigas* [8, 9], and *Laternula truncate* [10]. Due to the extensive studies on the visual ability of scallops (as a representatives of bivalves), many aspects of their vision are well understood, such as the identification of light-sensitive species [11], eye structure [12,13], eye function [3] and the environmental factors that impact on vision [14]. The light sensitive organ of scallops—described as a ‘pallial eye’—is located on the mid-mantle fold and functions as an alarm, to alert the animal to danger [3].

Bivalves with non-structured eyes can sense light, probably using dermal sensors [15]. Both small (< 15 mm) and large (> 15 mm) of *Limnoperna fortunei* show negative phototaxis and positive geotaxis when exposed to light [16]. *Dreissena polymorpha* also shows strongly negative phototaxis [2]. This behaviour might lead to selecting deeper and more sheltered sites, which are better protected against predators and dislodgement [4]. The exposure of *Mya arenaria* to light results in an interesting phenomenon: firstly retracts its paired siphons, then slowly expands them, and remains in this state during the remainder of the exposure [5].

The aim of the present study was to determine whether adult Pacific oysters were sensitive to light. Light sensitivities in larvae of some bivalve species have been confirmed, including *Mytilus sp*. [17] and oyster [18], but there is still uncertainty regarding the light sensitivity of the adult oysters. Adult oysters are sessile so light sensitivity is unlikely to have a significant impact on their movement to other locations. It may, however, play an important role in growth, reproduction, and antipredatory behavior.

## Materials and Methods

### Animal preparation

We obtained a number of adult Pacific oysters (shell height: 5–10 cm) from aquaculture farms located in the following areas: 66 from Yantai (China); 66 from Rushan (China); and 68 from Penglai (China). The oysters were cleaned with a brush and cultured in aquaria (80×40×40 cm, length×width×height) at Ludong University with water depth of 20 cm, temperature of 22°C, and salinity of 30. Air was pumped into aquaria throughout the 24 h period and feeding (with spirulina powder) took place three times per day. The oysters sampled from each location were separated into a treated group and a control group of equal numbers.

### Ethics Statement

No specific permissions were required for above sampling locations, Pacific oyster is not an endangered or protected species, and not vertebrate. The oysters used in this study were farmed.

### Flashlight experiments

Twenty-four hours prior to the start of the experiment, the oysters were randomly placed, one at a time, into a small transparent-glass aquarium (30×20×15 cm, length×width×height) through which air was continually pumped. Oysters were fed with spirulina powder. During the experiment, the ambient light condition and the light intensity of the oysters getting from the LED flashlight were detected by one luminometer for three times, respectively. The maximums and the minimums were chosen to determine the possible ranges of light intensity in above two locations as 141–154 lux and 5200–5300 lux. For the oysters in treated group, ‘Oyster activity periods’ were measured as follows: when the oyster shells opened, the camera initiated a video recording; a flashlight was turned on and then used to shine light (through the transparent glass for a 40 s period) onto the oysters, from a distance of about 5 cm; the flashlight was then turned off and quickly removed; the same camera was used to record the entire activity process. For oysters in control group, the flashlight was used without batteries to simulate the above experimental procedures including turning it on and off in case the associated sound or other factors could affect oyster behavior.

We examined two types of behavioural responses to light: (1) increased shell opening after turning the light on; and (2) closing the shell within 5 seconds after turning the light off. The oysters responding to light were classified into three groups: individuals not only opening their shells after turning the light on but also closing their shells after turning the light off (OC), individuals opening their shells after turning the light on (O), and individuals closing their shells after turning the light off (C).

### Statistical Analysis

The difference in the numbers of oysters responding to light between the treated group and the control group was tested using the Fisher exact test for each sampling location.

## Results

**Fig 1:**
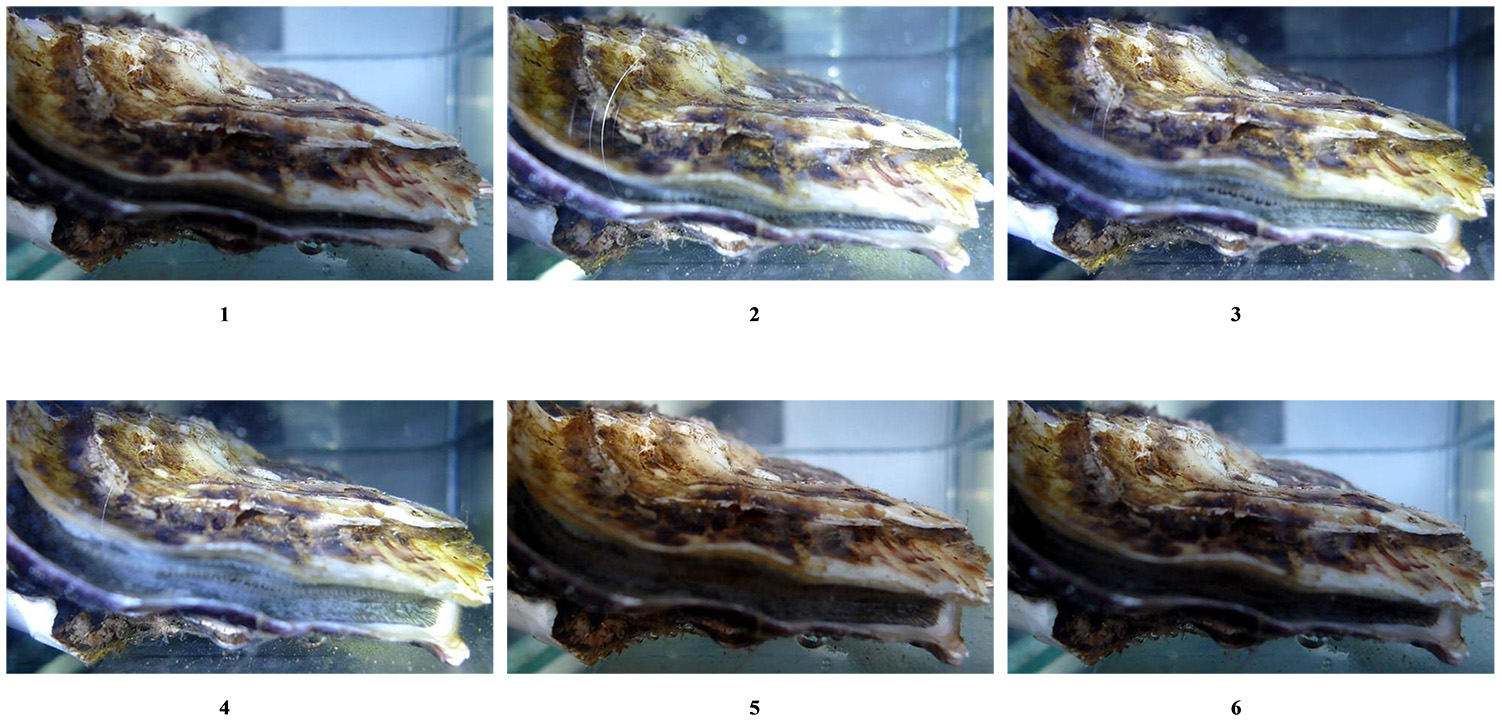
Responses of oysters to turning the flashlight on and off. 1, oyster filtering seawater with a slightly-opened shell prior to switch-on; 2, oyster immediately after switch-on; 3, oyster beginning to open its shells during switch-on; 4, oyster with a wider shell opening during switch-on; 5, oyster beginning to close its shell following switch-off; 6, reduction in size of shell opening following the disappearance of flashlights for several seconds. The ‘switch-on’ indicates the time when the flashlight is switched on; ‘switch-off’ indicates the time when the flashlight is switched off.

Some oysters responded to light when the flashlight was shone on them while they were filtering the seawater by increasing the size of the gap between the valves constantly and slowly to a larger or lesser extent. When the flashlight was switched off (Fig 1) some oysters responded by dramatically reducing or completely closing the gap between the valves. No oyster in the control groups showed such responses. A video of an oyster responding to light as example has been presented in S1 Video. The treated and control group differed significantly from each other in their responses to light, regardless of the sampling location (Table 1).

**Table 1:** The numbers of oysters responding to light in treated and control groups.

	Yantai	Rushan	Penglai	Total
The treated group (OC/T)	18/33	11/33	15/34	44/100
The control group (OC/T)	0/33	0/33	0/34	0/100
*P*-value	< 0.0001	< 0.0004	< 0.0001	< 0.0001
The treated group (O/T)	18/33	14/33	16/34	48/100
The control group (O/T)	0/33	0/33	0/34	0/100
*P*-value	< 0.0001	< 0.0002	< 0.0001	< 0.0001
The treated group (C/T)	21/33	18/33	19/34	58/100
The control group (C/T)	0/33	0/33	0/34	0/100
*P*-value	< 0.0001	< 0.0001	< 0.0001	< 0.0001

Note: OC, the number of individuals not only opening their shells after turning the light on but also closing their shells after turning the light off; O, the number of individuals opening their shells after turning the light on; C, the number of individuals closing their shells after turning the light off; T, the total individual number from any sampling site.

## Discussion

In this study, the extent of shell opening increased in some oysters when the oysters were exposed to light, and reduced when the light was turned off, The significant statistical differences between the numbers of responding oysters in the treated groups and the lack of such individuals in the controls suggests that adult Pacific oysters are sensitive to light The eyes of scallops and Ark clams are arranged along the mantle [3,19], those of some bivalves with dermal light sense were located in the skin, mantle or siphon [15]. Further investigation is required to locate the light sensitive organs in oysters.

Speiser and Johnsen thought that an explicit criterion for the occurrence of valve opening was “Mantle gapes were scored as open if there was a gap in the anterior mantle folds and the gills were exposed; mantle gapes were scored as closed if no gap was visible between the anterior mantle folds and the gills were not exposed” [20]. This is a static criterion. The degree of oyster valve opening is smaller than that of scallop valve opening and their gills are not exposed even at maximal opening. So, in our study, the criterion of sensing light was that the movements of opening or closing shells were visible, which was a dynamic criterion.

Following settlement and metamorphosis, oysters have a sessile lifestyle [21]. It has been previously assumed that the adult oyster did not need the ability to sense light, because they were able to filter algae without seeing the food. Nevertheless, when the oyster filters seawater to obtain food, its shell must be opened, although to a smaller extent than is the case for many other bivalves. During this period the oyster may also be vulnerable to attack by predators. In this context, the perception of dark, indicating the possible approach of predators, could trigger a reduction of the width of the shell opening, or a complete closure of the shell, to avoid danger. In our experiment, a cessation of light caused a darkening effect, to which oysters responded in a similar way. From these results we hypothesise that light-sensitive ability could be useful, and even important, to adult oysters, as a predation-avoidance mechanism.

Other mobile bivalves who can dig themselves into the sand with great speed to escape predation [22], or can swim by opening and closing their valves rapidly [23, 24], oysters had no choice but could only close their shells, which was the first step for survival. Some predators can crush or open the closed shells [25–28]. So, the second step for survival was to evolve the spiny and tough shells to do further defense against the predators. Other sessile bivalves may have the same mechanism, such as Chrmidae and Spondylidae.

Almost half of experimental oysters opened shells after turning the light on. This contrasts with *Dreissena polymorpha* which avoids illumination by moving to deeper and sheltered sites [4]. The reason why these molluscs respond differently to light is an interesting topic in the future. Perhaps oysters might open their valves wider to filter more food if illuminated seawater contains more algae [29].

## Conclusions

In this study, we obtained the preliminary evidence that the adult Pacific oyster may have light-sensitivity, which will stimulate more detailed research in oyster ethology and may be helpful to the husbandry and management of oysters.

## Supporting Information

S1 Video Video of oyster responding to light. (MP4)

## References

[1] Serb JM, Eernisse DJ. Charting evolution’s trajectory: Using molluscan eye diversity to understand parallel and convergent evolution education and outreach. Evolution. 2008; 1: 439–447.

[2] Matthew BT, Declan M, Marsden JE. Factors affecting the movement of adult zebra mussels (Dreissena polymorpha). J N Am Benthol Soc. 2002; 21: 468–475.

[3] Morton B. The evolutionary biology of the Bivalvia: The function of pallial eyes within the Pectinidae, with a description of those present in Patinopecten yessoensis. Evolutionary Biology of the Bivalvia Geological Society of London. 2000: 252–253.

[4] Kobak J, Nowacki P. Light-related behaviour of the zebra mussel (Dreissena polymorpha, Bivalvia). Fundam Appl Limnol. 2007; 169: 341–352.

[5] Selig H. Sensory equilibrium and dark adaptation in *Mya arenaria* . J Cell Biol. 1919; 5: 545–558 10.1085/jgp.1.5.545 PMC2140329 19871769

[6] Morton B. The pallial eyes of Ctenoides floridanus (Bivalvia: limoidea). J Molluscan Stud. 2000; 66: 449–455.

[7] Morton B, Peharda M. The biology and functional morphology of Arca noae (Bivalvia: Arcidae) from the Adriatic Sea, Croatia, with a discussion on the evolution of the bivalve mantle margin. Acta Zool. (Stockh). 2008; 89: 19–28.

[8] Wilkens LA. Hyperpolarizing photoreceptors in the eyes of the giant clam Tridacna-physiological evidence for both spiking and nonspiking cell-types. Comp Physiol. 1988; 163: 73–84. 10.1007/BF00611998 3385670

[9] Fankboner PV. Siphonal eyes of giant clams and their relationship to adjacent zooxanthellae. Veliger. 1981; 23: 145–249.

[10] Adal MN, Morton B. The fine structure of the pallial eyes of Laternula truncata (Bivalvia: Anomalodesmata: Pandoracea). Zoology. 1973; 170: 533–556.

[11] Land MF. Image formation by a concave reflector in the eye of the scallop, *Pecten maximus* . J Physiol. 1965; 179: 138–153. 5854374 10.1113/jphysiol.1965.sp007653 PMC1357328

[12] Barber VC, Evans EM, Land MF. The fine structure of the eye of the mollusc *Pecten maximus* . Z Zellforsch Mikrosk Anat. 1967; 76: 25–312. 5590644

[13] Malkowsky Y, Jochum A. Three-dimensional reconstructions of pallial eyes in Pectinidae (Mollusca: Bivalvia). Acta Zool. (Stockh). 2015; 96: 167–173.

[14] Malkowsky Y, Götze M-C. Impact of habitat and life trait on character evolution of pallialeyes in Pectinidae (Mollusca: Bivalvia). Org Divers Evol. 2014; 14: 173–185.

[15] Ramirez MD, Speiser DI, Pankey MS, Oakley TH. Understanding the dermal light sense in the context of integrative photoreceptor cell biology. Vis Neurosci. 2011; 28: 265–279. 10.1017/S0952523811000150 21736861

[16] Yumiko U, Keiji I, Masmi H. Laboratory experiments on behaviour and movement of a freshwater mussel, *Limnoperna fortunei* . J Molluscan Stud. 1996; 62: 327–341.

[17] Bayne BL. The responses of the larvae of *Mytilus edulis* L. to light and gravity. Oikos. 1964; 15: 162–174.

[18] Magalhães TR, Neves RA, Valentin JL. Do the changes in temperature and light affect the functional response of the benthic mud snail Heleobia australis (Mollusca: Gastropoda)?. An Acad Bras Cienc. 2012; 86: 1197–1206. 10.1590/0001-3765201420130093 25014915

[19] Nilsson Dan-E. Eyes as optical alarm systems in fan worms and ark clams. Philos T R Soc B. 1994; 346: 195–212.

[20] Speiser DI, Johnsen S. Scallops visually respond to the size and speed of virtual particles. J Exp Biol. 2008; 211: 2066–2070. 10.1242/jeb.017038 18552295

[21] Zhang G, Fang X, Guo X, Li L, Luo R, et al The oyster genome reveals stress adaptation and complexity of shell formation. Nature. 2012; 490: 49–54. 10.1038/nature11413 22992520

[22] https://en.wikipedia.org/wiki/Bivalvia#cite_note-72.

[23] Brokordt KB, Himmelman JB, Guderley HE. Effect of reproduction on escape responses and muscle metabolic capacities in the scallop Chlamys islandica Muller 1776. J Exp Mar Bio Ecol. 2000; 251: 205–225. 10960615 10.1016/s0022-0981(00)00215-x

[24] Laming SR, Jenkins SR, McCarthy ID. Repeatability of escape response performance in the queen scallop, *Aequipecten opercularis* . J Exp Biol. 2013; 216: 3264–3272. 10.1242/jeb.080416 23685972

[25] Hulscher JB. The oystercatcher Haematopus ostralegus as a predator of the bivalve Macoma balthica in the Dutch Wadden Sea. Ardea. 1982; 70: 89–152.

[26] Agnar Ingolfsson, Estrella Bruce T. The development of shell-cracking behavior in herring gulls. The Auk. 1978; 95: 577–579.

[27] Hall KRL, Schaller GB. Tool-using behavior of the California sea otter. J. Mammal. 1964; 45: 287–298.

[28] Fukuyamaa AK, Olivera JS. Sea star and walrus predation on bivalves in Norton Sound, Bering Sea, Alaska. Ophelia. 1985; 24: 17–36.

[29] Cheirsilp B, Torpee S. Enhanced growth and lipid production of microalgae under mixotrophic culture condition: effect of light intensity, glucose concentration and fed-batch cultivation. Bioresour Technol. 2012; 110: 510–516. 10.1016/j.biortech.2012.01.125 22361073

